# Post-Marketing Surveillance of Adverse Events for the Recombinant Zoster Vaccine Among the Population over 50 Years Old in Hangzhou, China

**DOI:** 10.3390/vaccines12121376

**Published:** 2024-12-06

**Authors:** Jing Wang, Jian Du, Yan Liu, Yuyang Xu, Jiayin Han, Xuechao Zhang

**Affiliations:** 1 Department of Infectious Disease Control and Prevention, Hangzhou Center for Diseases Control and Prevention (Hangzhou Health Supervision Institution), Hangzhou 310021, China; wangjing@hzcdc.com.cn; 2 Department of Expanded Program on Immunization, Hangzhou Center for Disease Control and Prevention, Hangzhou 310021, China; dujian@hzcdc.com.cn (J.D.); xuyuyang@hzcdc.com.cn (Y.X.); hanjiayin@hzcdc.com.cn (J.H.); xchzhang@hzcdc.com.cn (X.Z.)

**Keywords:** adverse events following immunization, recombinant zoster vaccine, vaccine safety

## Abstract

Objectives: This study aimed to evaluate the safety profile of the recombinant zoster vaccine (RZV) after its marketing in China. Methods: We present a descriptive analysis and safety signal assessment of adverse events following immunization (AEFI) associated with RZV between September 2020 and December 2023. The descriptive data collected includes demographic characteristics and the classification of characteristics of AEFI cases, while vaccine safety signal assessment was evaluated using the reporting odds ratio (ROR). Results: In total, we documented 275 AEFI cases following RZV vaccination, with a reporting rate of 76.22/10,000 doses administered. Notably, only one case was classified as serious, and the reporting rates were significantly higher among females, individuals aged 50–59 years, and those residing in rural areas. Furthermore, the reporting rate for the first dose exceeded that for the second dose. Among the reported AEFI cases, 98.91% were attributed to vaccine product-related reactions, and 97.45% were initially reported by either the vaccine recipient or their guardians. The interval between vaccination and symptom onset was predominant within 3 d after vaccination. The disproportionality analysis identified five positive signals—fever (37.5–38.5 °C), injection site reactions greater than 5 cm, pain, Henoch Schönlein purpura (HSP), and swelling—which suggests a stronger association with the RZV than the expected threshold. Conclusion: In summary, RZV demonstrated a favorable safety profile. However, continued monitoring and research on the long-term safety implications of RZV are needed.

## 1. Introduction

Herpes zoster (HZ) is a neurological condition that primarily results from the reactivation of the varicella zoster virus. This virus remains dormant in the sensory ganglia of the host. However, in individuals with compromised immune systems, HZ is more likely to develop. Notably, individuals aged ≥50 have an increased risk of developing postherpetic neuralgia (PHN) following HZ, with symptoms possibly persisting for several months [1,2]. Findings from a systematic literature review of 69 studies revealed that the incidence of HZ ranges from 5.23 to 10.9 cases per 1000 person-years, with a higher prevalence observed in females than in males. Furthermore, the incidence of HZ notably increases with advancing age and over time [3], particularly for those with chronic underlying conditions [4,5].

Vaccination is the primary strategy for preventing HZ [6]. Currently, there are two widely used HZ vaccines: the live zoster vaccine (ZVL) and recombinant zoster vaccine (RZV). The former was approved in 2006, whereas the latter received authorization for marketing only in 2017 in the USA [7]. Both vaccines have been shown to effectively reduce the incidence of HZ [7,8]. Furthermore, the efficacy of both vaccines in individuals aged ≥50 was found to be 46% and 85%, respectively, based on findings from a retrospective analysis of real-world effectiveness data gathered over 10 years [7]. From a cost-effectiveness standpoint, the incremental cost-effectiveness ratios (ICERs) for RZV were higher than those for ZVL, with values ranging from USD 22,097 to USD 27,486 per quality-adjusted life year (QALY) in North America [9] and from USD 3428 to USD 5743 per QALY in China [10]. Additionally, various countries have incorporated RZV into their national immunization programs because it can be used for patients with compromised immune function and immune suppression, whereas ZVL is contraindicated in this population [7,11].

Nevertheless, in some countries, such as Saudi Arabia, RZV use among older adults remains relatively low, primarily because of the potential side effects associated with the vaccination [4]. Therefore, the adverse events following RZV vaccination have attracted substantial attention from the research community. A network meta-analysis of randomized controlled trials revealed that the association between adverse events and RZV was statistically stronger than that with ZVL or placebo [12]. Hence, the Centers for Disease Control and Prevention (CDC) and Food and Drug Administration (FDA) initiated the passive safety monitoring of the recombinant zoster vaccine after it was authorized for marketing in the United States. Furthermore, Hesse et al. 2019 conducted an observational study. They reported that the rate of adverse events associated with RZV was approximately 13.69 per 10,000 doses, with common signs and symptoms, such as fever and injection site reactions [13]. Their findings were consistent with those observed in premarketing clinical trials, with serious adverse events occurring in 3% of patients [13].

In addition, the RZV, developed by GlaxoSmithKline, was approved by the National Medical Products Administration of China in June 2020 for the prevention of shingles in adults aged ≥50 years, priced at approximately USD 230 per dose [14]. Moreover, a Phase IV observer-blind study was conducted in China between 2021 and 2023. The results revealed that adverse reactions occurring within 7 d post-vaccination were significantly more frequent in the RZV group (76.3%) than in the placebo group (14.4%). However, the most commonly reported local and systemic adverse events were pain and fatigue [15].

Hangzhou, a city located in the southeastern region of China, is regarded as one of the country’s most advanced cities in the country. Following the introduction of RZV in Hangzhou in September 2020, the increasing vaccination rate received considerable public attention due to the vaccine’s relatively high cost and the particular characteristics of the demographic being targeted. Considering the limited safety monitoring data available for the RZV following its approval in China, we used data from the National Adverse Events Following Immunization (AEFI) Information System of China to systematically investigate AEFI associated with the RZV in Hangzhou over the past three years. We compared these findings with the reported AEFI associated with other vaccinations during the same period, aiming to evaluate the safety of RZV use among the target population in China and to explore the potential underlying factors.

## 2. Materials and Methods

### 2.1. Data Source

Data were sourced from the Chinese National AEFI Information System (CNAEFIS), a passive surveillance system established for monitoring vaccine safety that has been in operation since 2008 [16]. All healthcare institutions, vaccination clinics, Centers for Disease Control and Prevention (CDC), vaccine manufacturers and distributors, and vaccine recipients (or their guardians) were responsible for reporting the incidence of AEFI cases that emerged after vaccination. The incidents and cases were systematically collected by the CDCs and reported to the CNAEFIS through an online platform. A specialized AEFI investigation team, made up of experts from clinical medicine, epidemiology, pharmacy, and adverse drug reactions (ADR), evaluated the causal relationship between these incidents and vaccination, classifying them according to the World Health Organization (WHO) manual [17]. The results of the evaluation and classification were also updated in CNAEFIS. Furthermore, data on zoster vaccine administration were sourced from a provincial immunization registration system, which was established in 2005 and operated on the internet.

### 2.2. Data Extraction

Shingerix^®^, is a recombinant zoster vaccine that is approved for use in mainland China. It was produced by GlaxoSmithKline (GSK) located in Belgium. Its price is at approximately USD 230 per dose in China, and it is suitable for people for our target population. The immunization regimen comprised two doses (0.5 mL) initially, with the second dose administered two months after the first.

Data on recipients of the RZV extracted from the provincial immunization registration system comprised individuals vaccinated between 1 September 2020 and 31 December 2023. The AEFI reported during this period were retrieved from the CNAEFIS database based on the onset dates. The extracted data included demographic information (sex, age, and residential area), vaccination-related details (vaccination date, number of doses administered, onset date, reporting date, initial reporting source, the interval between vaccination and onset), and diagnostic information related to the AEFI cases (diagnosis classification by cause-specific criteria and severity).

### 2.3. Data Analysis

The database was systematically compiled and organized using Microsoft Excel (2019), and the data were analyzed using chi-square utilizing the Statistical Package for Social Sciences (SPSS, Version 22.0). We categorized the cases by age based on global clinical studies on adverse reactions outlined in the vaccine’s instructions for use. Additionally, we classified cases according to the time interval from vaccination to symptom onset, following China’s national AEFI guidelines.

Descriptive statistics included the analysis of the temporal distribution (by month), residential area (categorized into urban, suburban, and rural areas), sex (sorted by three age groups), dose number, cause-specific classification, initial reporting source, and time interval of AEFI occurrence. We calculated the AEFI reporting rate for each variable as the total number of AEFIs reported for that variable during the study period divided by the total number of doses administered for that variable and expressed per 10,000 doses. Quantitative data were presented as numerical values and/or rates, and the differences were analyzed using the chi-square test. Differences in sex after classification were analyzed using stratified chi-square test. Statistical significance was set at *p* < 0.05.

We used the disproportionality analysis approach to detect concerns of potential vaccine safety in this study. This method flags a safety signal when the observed link between a specific vaccine and a diagnosis exceeds the expected threshold, which indicates a disproportionate relationship. We used the reporting odds ratio (ROR) as our analytical tool, and a signal was considered positive if the lower limit of the 95% confidence interval (CI) for the ROR was greater than one [18,19,20]. None of the cases reported in this study had records of simultaneous administration of multiple vaccines. However, only the primary diagnosis was used in this report, considering that patients with AEFI may present with multiple concurrent diagnoses.

### 2.4. Study Ethics

The research was performed following the principles of the Declaration of Helsinki (1964) and was approved by the Ethics Committee at the Hangzhou Center for Disease Control and Prevention (protocol numbers 2023-KS10, dated 8 December 2023). All data used in the analysis had no personal identifiers, ensuring that only anonymized data were used.

## 3. Results

### 3.1. Descriptive Results

**Figure 1:**
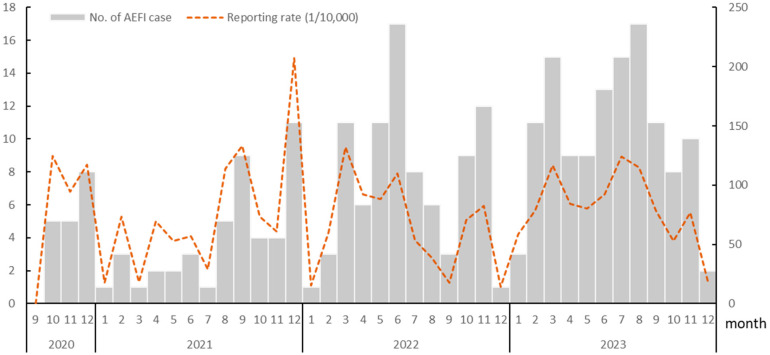
Number of adverse events following immunization cases and reporting rate (per 10,000 doses) associated with recombinant zoster vaccine between 2020 and 2023 in Hangzhou, China.

Since the recombinant zoster vaccine was first administered in Hangzhou, China, in September 2020, approximately 36,079 doses have been administered to the entire city as of 31 December 2023, with an average of 902.0 doses administered per month. Excluding the first month, during which no AEFI cases were reported, monthly reports of AEFI associated with the RZV were recorded for the subsequent 39 months, totaling 275 cases and with a reporting rate of 76.22 per 10,000 doses. Of these cases, only one serious AEFI was reported in November 2023, with a serious AEFI reporting rate of 0.28 per 10,000 doses. The remaining 274 patients had nonserious diseases. Furthermore, the number of RZV vaccinations showed a steady annual increase between 2020 and 2023. This increase culminated in 2023, with 14,759 doses administered across the city. Concurrently, the number of AEFI reports increased, rising from 18 cases in 2020 to 123 cases in 2023; however, the reporting rate did not significantly differ over the four-year period (χ^2^ = 5.10 *p* = 0.17) (Table 1 and Figure 1).

All vaccine recipients were aged ≥50 years, with the 50–59 age group receiving the highest number of doses (19,208 doses). Approximately 170 AEFI cases were reported in this group, with a reporting rate of 88.50 per 10,000 doses. This rate was also the highest among the three age groups. The number of vaccinations administered to the subsequent age group (60–69 years) decreased to 13,198 doses, with 86 reported AEFI, which resulted in a reporting rate of 65.16 per 10,000 doses. Those in the ≥70 years age group showed the lowest numbers in terms of vaccine doses, AEFI cases, and reporting rates. The reported numbers reported in this group declined to 3673 doses, 19 cases, and 51.73 per 10,000 doses. Notably, the three age groups significantly differed (χ^2^ = 8.88 *p* < 0.05). Upon stratification by age groups, we discovered that the reporting rate of AEFI was highest among women aged 50–59, and the lowest among men aged ≥70 years. The vaccination and reporting rate of AEFI were both higher among women in all three age groups than in men. Through stratified chi-square analysis, a statistically significant difference in AEFI incidence was observed between men and women (χ^2^ = 14.13, *p* < 0.05). Furthermore, the first and second doses administered were relatively comparable, which were 19,790 and 16,289 doses, respectively, indicating that the majority of individuals were fully vaccinated. However, the numbers of AEFI associated with the two doses significantly differed, with 168 cases reported for the former (reporting rate of 106.62 per 10,000 doses) and only 87 cases for the latter (reporting rate of 39.29 per 10,000 doses) (χ^2^ = 53.55 *p* < 0.05).

In terms of geographical distribution, the Central City recorded the highest number of varicella vaccine doses administered (23,626) and concurrently reported the highest number of varicella vaccine-related AEFI cases (191). However, those in the suburbs received 9231 doses, resulting in 39 reported cases of AEFI, whereas rural areas received the fewest doses during the study period (3222), with a total of 45 reported AEFI cases. On the other hand, with respect to AEFI reporting rates, remote areas had the highest rate (139.66 per 10,000 doses), followed by central urban areas (80.84 per 10,000 doses), with near central urban areas recording the lowest rate (42.25 per 10,000 doses). The vaccination rates significantly differed among these three areas (χ^2^ = 20.28 *p* < 0.05).

Following vaccination, 98.91% of the AEFI cases were classified by the diagnostic panel as vaccine product-related reactions (272 cases), which included one case of a serious reaction, representing 98.91% of the total. Furthermore, these three cases were classified as coincidental events. Most of the initial reports (268; 97.45%) originated from the vaccine recipients themselves or their guardians, whereas only seven cases were reported by vaccination clinics. Regarding the time interval of AEFI occurrence, 257 cases (93.45%) occurred within 24 h post-vaccination, whereas 11 cases (4.00%) were reported within 2–3 days after vaccination. However, only seven cases (2.55%) occurred beyond 4 d after vaccination (Table 2).

### 3.2. Clinical Diagnosis

Table 3 shows 275 AEFI cases reported after receiving the RZV. Among them, 274 cases were mild vaccine-related reactions. Based on the diagnostic results, fever was the most reported diagnosis, accounting for 162 cases (58.91%). Among these cases, those with a temperature ranging from 37.5–38.5 °C (123 cases, 34.09 per 10,000 doses) were markedly higher than those having ≥38.5 °C (39 cases, 10.81 per 10,000 doses). The second most commonly reported diagnosis was an injection site reaction, characterized by redness, swelling, and induration at the injection site, observed in 83 cases (30.18%). This reaction was divided into three grades based on the size of the affected area, which was ranked based on the number of cases: 2.6–5.0 cm (45 cases), >5.0 cm (27 cases), and ≤2.5 cm (11 cases). There were nine cases of fatigue and six cases of pain, of which one was classified as coincidental. Furthermore, there were four cases of allergic rash and three cases of other types of rashes, of which one was classified as coincidental. Additionally, gastrointestinal reactions, swelling, and neurological reactions were each observed in two instances of AEFI after vaccination. Only a single case of cardiovascular disease was reported; however, this case was subsequently determined by the expert panel to be coincidental. The only case of a serious vaccine-related reaction diagnosed was Henoch–Schönlein purpura (HSP).

From the positive signals, the following were identified: fever ranging from 37.5 °C to 38.5 °C (ROR-1.96, SE: 6.09), swelling (ROR-1.96, SE: 5.02), HSP (ROR-1.96, SE: 2.73), reaction at the injection site (>5.0 cm) (ROR-1.96, SE: 2.57), and pain (ROR-1.96, SE: 1.86).

## 4. Discussion

RZV is specifically formulated for adults aged ≥50 years to prevent shingles and its associated complications. This vaccine stimulates the human immune system to generate antibodies, thereby achieving its preventive objective [7]. It was launched in China in 2020 and has substantially contributed to making shingle vaccination available for our target population, with an increasing acceptance rate observed annually. However, all vaccinations carry inherent risks, including local and systemic reactions. The occurrence of AEFI impacts the health of recipients and may also adversely affect immunization programs. Therefore, the timely and accurate monitoring and evaluation of AEFI associated with RZV are necessary to maintain public confidence and ensure the success of immunization initiatives.

An analysis of RZV-associated adverse events reported in the NAEFISS system revealed that the reporting rate of AEFI for the RZV in Hangzhou was 72 per 10,000 doses. Moreover, RZV has been available in the market for over 3 years, and this rate is significantly higher than both the reporting rate documented by GSK (16.82) [21] and the passive monitoring results from the United States 2 years after the vaccine was launched (13.69 per 10,000 doses) [13]. Furthermore, when compared with existing research findings within China, the AEFI reporting rate following RZV vaccination in Hangzhou was considerably greater than that observed by Bo et al. 2022 in Guangdong Province 1 year after the vaccine was introduced (19.67 per 10,000 doses) [14]. The Weber effect may explain the elevated reporting rate observed. The Weber effect posits that the incidence of reported adverse events tends to increase shortly after a drug’s approval before gradually stabilizing [19]. In addition, it is important to consider the influence of large-scale COVID-19 vaccination campaigns conducted between 2020 and 2023 because these campaigns may have significantly heightened public awareness regarding adverse reactions following vaccination [22].

The demographic information of the population reporting RZV-related adverse event reports indicated that there was a significant age group disparity in the reporting rate of AEFI, with the highest rate occurring in the 50–59 age group, aligning with the findings of Ramona’s team and Tavares-Da-Silva’s team [11,21]. This difference was also observed in vulnerable populations [23,24]. After sex was sorted by different age groups, we discovered that the sensitivity of females to adverse events was higher than that of males in all age groups, including 50–59, 60–69, and ≥70 years. Similar outcomes were also reported in a review of adverse reactions to COVID-19 vaccines [25]. However, reporting bias between sex and age groups may have influenced this result. Additionally, the results may be attributed to the biological distinctions between males and females, including hormones, genes, and past exposure history [26]. Moreover, disparities in the AEFI reporting rates were observed across different residential areas, with rural areas showing the highest rates. This phenomenon may be attributed to the lower level of vaccine knowledge among older adults in these areas and their insufficient confidence in vaccine safety [27].

The findings of this study reveal that the reporting rate of AEFIs after the first dose was higher than that following the second dose, with the majority of AEFIs occurring within 0–1 d post-vaccination. This observation aligns with the results reported in Australia and Guangdong Province of China, after the introduction of the vaccine [11,14]. However, the two doses did not significantly differ in clinical trials involving immunocompetent adults aged ≥50 years across 18 countries, including those in Europe, Latin America, and the Asia-Pacific region [28,29,30]. Notably, the difference between the two, in the real world, may be due to the fact that recipients of the first dose paid more attention to adverse reactions and hence reported higher rates of occurrence and that recipients who experienced adverse reactions after the first dose did not receive the second dose. However, in frail populations, researchers have found that medications were used more often for treatment after the second dose than after the first (28% vs. 13%) [23]. Regarding the time interval from vaccination to the onset of AEFI, a significant majority of AEFIs occurred within 3 days (97.45%), which aligns with the post-marketing surveillance findings in the United States and Europe [13,21]. A plausible explanation for this observation is that the interval between vaccination and the most frequently reported AEFIs (such as fever or injection site reactions) is relatively brief because the observation may arise from local inflammation or dysregulation of central temperature control [19]. Seven cases showed an onset time and interval exceeding 4 d, which were all generally mild reactions (primarily rashes and pain, among others). These onset times were influenced by the considerable time spent between reporting and vaccination, resulting in some recall bias. There was one case that occurred in which a rash was caused by other reasons but not associated with vaccination. After discussion by the AEFI diagnostic panel, this event was categorized as a coincidence. Similar findings of delayed skin rashes were reported in studies on adverse reactions following COVID-19 vaccination, where T-cell-mediated hypersensitivity plays a major role in the pathogenesis of these skin lesions [25], suggesting that these reactions are neither novel nor unidentified safety concerns of vaccination.

In addition, our study revealed a notable finding that most of the initial sources of report (97.45%) were vaccine recipients or guardians. This indicates that most reported AEFI cases are not immediate post-vaccination events; rather, they are identified and reported by the recipients or guardians after returning home. However, only seven cases (1.94%) occurred during the post-vaccination observation period. These cases were documented in the vaccination clinic’s reporting system. These findings suggest that serious anaphylactic reactions (such as anaphylactic shock and laryngeal edema) are exceedingly rare following RZV vaccination.

In our study, the most frequently diagnosed AEFI was fever (162 cases, 58.91%), with suspected positive signals observed for mild to moderate fever ranging from 37.5 to 38.5 °C, indicating that this symptom showed a higher reporting rate than the average levels of other vaccines administered during the study period [13,21]. Another commonly reported positive signal was a large injection site reaction, primarily characterized by redness and induration exceeding 5 cm in diameter. This finding aligns with results from a study published in the New England Journal of Medicine [28,29]. They reported that approximately 17% of participants had erythema and induration > 3.5 inches, which was typically self-limiting; however, it resolved within several days [13]. The reaction at the injection site could have resulted from the stimulation of nociceptive sensory neurons during vaccination or by a subsequent inflammatory process in the damaged tissue [19]. Pain, the third positive signal in this study, has also been mentioned in multiple research findings [15,23]. Moreover, the AS01_b_ adjuvant may significantly influence these three symptoms [31]. The reported incidence of local and systemic adverse reactions associated with RZV was notably higher than that of other traditional vaccines such as hepatitis B and influenza vaccines. This increased incidence may be a result of the use of the patented AS01_b_ adjuvant in the Shingrix HZ vaccine, which is a liposome-based complex adjuvant system containing two immune stimulants: monophosphoryl rapid A and saponin QS21 [14]. This adjuvant system can simultaneously induce robust humoral and cellular immune responses in the host [32]. Furthermore, research indicates that AS01 is rapidly distributed to the muscle and lymph nodes within 30 min after intramuscular injection, prompting a swift influx of neutrophils, monocytes, dendritic cells (DCs), and T cell populations into both the injection site and the lymph nodes [33]. Consequently, this novel adjuvant enhances immunogenicity and results in more frequent general reactions [7,31].

Two additional positive signals were identified during the diagnosis of HSP and swelling. HSP is a systemic vasculitis resulting from a hypersensitivity reaction that affects small blood vessels. Notably, vaccine-associated cases are relatively rare [31]. In this study, only one case of HSP associated with vaccination was reported, which was classified as a severe case following the WHO Health Organization criteria because of the necessity for hospitalization [17]. Instances of HSP events have been documented following vaccinations against influenza, hepatitis B, and COVID-19 [34,35,36]. However, its cause remains unclear, potentially caused by vaccine antigens also attacking human proteins with similar structures through molecular mimicry, hence leading to an autoimmune disease. It may also be due to adjuvants in the vaccine binding to pattern recognition receptors, recruiting natural immune cells, and secreting large amounts of cytokines to induce innate immune responses [37]. However, based on the research findings of The Brighton Collaboration Fever Working Group, HSP primarily occurs in children aged 1–17, with fewer cases in older individuals (>65 years) [38]; therefore, the occurrence of this abnormal reaction after RZV vaccination requires more attention. We also identified two cases of swelling that occurred post-vaccination. This swelling was not at the injection site but in the facial region or lower limbs, which differs from the injection site swelling reported in studies conducted in the United States and Australia and is presumed to result from allergic reactions leading to increased vascular permeability, which allows fluid from the bloodstream to infiltrate the surrounding tissues. However, the precise etiology of this condition needs further investigation.

This study also has some limitations. First, data were obtained using a passive AEFI surveillance system. Furthermore, the surveillance systems themselves have some inherent limitations; for example, reporters may not report all adverse events due to concerns or may over-report certain reactions due to high attention to the new vaccine. Additionally, the lack of information regarding the recipient’s medical history, current illness, and medication use makes it difficult to determine the true incidence of adverse events. In this case, the passive AEFI surveillance system can only assess potential risks based on case data and provide suspicious safety signals. However, these initially identified signals still need to be verified and confirmed through further research to ensure the accuracy and reliability of the results.

## 5. Conclusions

Based on our surveillance data between September 2020 and December 2023, increased scrutiny may have caused a high reporting rate of AEFI for the newly introduced recombinant varicella vaccine; however, it has an overall good tolerability among individuals aged ≥50 years in China, with only one case of severe HSP documented. Therefore, continued vigilance and research are essential to assess the long-term safety of this vaccine among older adults who are Chinese.

## Figures and Tables

**Table 1:** Demographic characteristics of vaccination and adverse events following immunization cases following recombinant zoster vaccine in Hangzhou, China.

Characteristics			Number of Total Dose	Number of AEFI Case	Reporting Rate (per 10,000 Doses)	χ^2^	*p*-Value
Year	2020		1755	18	102.56	5.10	0.17
	2021		5919	46	77.72		
	2022		13,646	88	64.49		
	2023		14,759	123	83.34		
Age (Y)	50–59		19,208	170	88.50	8.88	<0.05
	60–69		13,198	86	65.16		
	≥70		3673	19	51.73		
Sex (sorted by age groups)	Male	50–59 years	5305	23	43.36	14.13	<0.05
		60–69 years	4867	30	61.64		
		≥70 years	1602	5	31.21		
	Female	50–59 years	13,903	145	104.29		
		60–69 years	8331	57	68.42		
		≥70 years	2071	15	72.43		
Dose number	1		19,790	211	106.62	53.55	<0.05
	2		16,289	64	39.29		
Residential area	central city		23,626	191	80.84	20.28	<0.05
	suburbs		9231	39	42.25		
	rural areas		3222	45	139.66		
Total			36,079	275	76.22		

AEFI, adverse events following immunization; *p* ≤ 0.05; χ^2^, Chi-square.

**Table 2:** Classification of characteristics associated with adverse events following immunization cases related to recombinant zoster vaccine in Hangzhou, China (N = 275).

Characteristics		Number of AEFI Case	Percentage (%)	Reporting Rate (per 10,000 Doses)
Cause-specific classification	Vaccine product-related reaction	272	98.91	75.39
	Coincidental event	3	1.09	0.83
Initial reporting source	Vaccination clinic	7	2.55	1.94
	Vaccine recipient/their guardian	268	97.45	74.28
Time interval of AEFI occurrence (days)	0–1	257	93.45	71.23
	2–3	11	4.00	3.05
	4–7	1	0.36	0.28
	8–14	3	1.09	0.83
	≥15	3	1.09	0.83
Total		275	100.00	76.22

**Table 3:** Clinical diagnosis of adverse events after immunization cases associated with recombinant zoster vaccine in Hangzhou, China (N = 275).

Clinical Diagnosis		Number of AEFI Cases			
		Vaccine Product- Related Reaction	Coincidental Event	Reporting Rate (per 10,000 Doses)	ROR-1.96SE *
Fever (°C)					
	37.5–38.5	123	0	34.09	6.09 **
Injection site reaction (cm)	≥38.6	39	0	10.81	0.78
	≤2.5	11	0	3.05	0.34
	2.6–5.0	45	0	12.47	0.54
	>5.0	27	0	7.48	2.57 **
Rash					
	Allergic	4	0	1.11	0.22
	Others	2	1	0.83	0.82
Pain ^#^		5	1	1.66	1.86 **
Fatigue		9	0	2.49	0.99
HSP ^##^ (serious case)		1	0	0.28	2.73 **
Gastrointestinal reactions		2	0	0.55	0.32
Swelling		2	0	0.55	5.02 **
Neurological reactions		2	0	0.55	0.57
Cardiovascular disease		0	1	0.28	0.94

* ROR: reporting odds ratio, SE: standard error. ** Positive signal. ^#^ This may include other types of pain, such as muscle and joint pain, as well as pain at the injection site with no other symptoms. ^##^ HSP: Henoch–Schönlein purpura.

## Data Availability

All data were unavailable because of privacy or ethical restrictions.
